# Work satisfaction among neuroradiology staff after receiving follow up reports of thrombectomy stroke patients

**DOI:** 10.1371/journal.pone.0251889

**Published:** 2021-05-19

**Authors:** Charlotte Hager, Homan Taufik, Friederike Blum, Andrea Stockero, Martin Wiesmann, Arno Reich, Rebecca May, Omid Nikoubashman

**Affiliations:** 1 Department of Neuroradiology, University Hospital RWTH Aachen, Aachen, Germany; 2 Department of Neurology, University Hospital RWTH Aachen, Aachen, Germany; Hospital Dr. Rafael A. Calderón Guardia, CCSS, COSTA RICA

## Abstract

**Background and purpose:**

During a period of 6 months, we provided our entire neuroradiological staff including physicians, radiographers, and researchers with systematic feedback via email on the further clinical course of stroke patients who underwent mechanical thrombectomy. We analyzed the effects of this feedback on work satisfaction, work meaningfulness and valuation of the therapy among our staff.

**Methods:**

Our staff completed two self-reported questionnaires before and after the period of six months with systematic feedback.

**Results:**

Employees with higher work meaningfulness and higher work satisfaction valuated endovascular stroke therapy as more useful (p<0.001). A good clinical outcome was regarded more motivating than a good interventional outcome (p<0.001). Receiving systematic feedback did not increase work satisfaction (p = 0.318) or work meaningfulness (p = 0.178). Radiographers valuated the usefulness of interventional therapy the worst of all employees (p≤ 0.017). After the feedback period, 75% of radiographers estimated stroke as a more severe disease than before. Also, their desire for feedback decreased significantly (p = 0.007). Primarily patient cases with unfavorable outcomes were remembered by the staff.

**Conclusions:**

Systematic email feedback does not per se enhance work satisfaction or work meaningfulness among employees. However, receiving feedback is educative for the staff. Evaluating work satisfaction and the perception of treatment may help to identify unexpected issues and may therefore help to find specific measures that increase work satisfaction and motivation.

## Introduction

Since thrombectomy became the standard treatment option for emergent large vessel occlusion (ELVO) stroke, neuroradiology became an increasingly interventional specialty [1–3]. At the same time, direct consultation and verbal communication between referring physicians and radiologists have been shown to decrease tremendously since radiology has become filmless through the implementation of electronic imaging systems (PACS, Picture Archiving and Communication System) [4–7]. In stroke and patient care in general, neuroradiology departments are usually completely out of touch with actual patient care. In 2013, a survey emphasized this minimal doctor-patient-contact by uncovering that only 54% of the patients examined in the radiology department realised that their examiner, the radiologist, was a physician [8]. Numerous previous studies established the advantage and value of clinical-radiologic rounds [5,6,9–12]. However, clinical-radiologic rounds are often difficult to implement in the existing hospital structure, as rounds are often time-consuming and time schedules of staff can vary from day to day [6,13]. That is why, when our neuroradiology staff expressed desire to receive more feedback about the patients after their therapy, we instead decided to conduct systematic clinical feedback via e-mail.

We hypothesized that such feedback would improve job satisfaction and subjective work meaningfulness among employees, because a previous survey confirmed that receiving helpful quality performance data, e.g. statistical analysis of patients’ outcomes, predicts a greater professional satisfaction [14].

The main aim of this study was to investigate the effects of systematic email feedback of endovascular treated stroke patients on work satisfaction, work meaningfulness and valuation of endovascular stroke therapy (EST) among radiology staff.

## Methods

### Questionnaire

Building off previous research and using a longitudinal design, we constructed two self-reported questionnaires consisting of 25 questions in the first version and 59 questions in version two, which were distributed before and after six months of systematic patient feedback (January and July 2020). Neuroradiology department employees were asked to anonymously and voluntarily answer questions addressing: demographic data (gender, work years, profession), work volition, wage satisfaction, subjective competence-assessment, valuation of EST and preference for patient feedback. The questionnaire also included the Work and Meaning Inventory scale by Michael F. Steger, a 10-item tool evaluating the subjective work meaningfulness. Steger and colleagues reported data confirming the scale’s validity and showed a high level of internal consistency with an alpha coefficient of 0.93 [15]. The instrument has been used in numerous previous studies of health-care employees [16–18]. Furthermore, we applied the 5-item Job Satisfaction Scale by Judge et al. Internal consistency of this scale has been proven high with Cronbach’s alpha of 0.8 and higher [19,20]. Responders had to rate the scales on a 5-point Likert scale with response options ranging from “I don’t agree at all” to”I completely agree” [15]. Responses were added to a total score, with higher scores always indicating higher work satisfaction and work meaningfulness. In the second version of the questionnaire we added questions concerning the career training-needs, motivational and demotivational factors for work, which cases specifically touched the employees emotionally, the feedback’s consequences, its influence on the work satisfaction and possible revision in the assessment of stroke-severity after receiving feedback.

The institutional ethics committee at the RWTH Aachen Faculty of Medicine has approved this study. Study participants voluntarily participated in this study and gave written informed consent for data analysis and use. The questionnaires were evaluated anonymously, so that no conclusions could be drawn about individual study participants.

### Feedback

Staff members received systematic feedback via email about the short-term (i. e. from admission to discharge) and long-term clinical outcome (i. e. 90 days after admission) of endovascular treated stroke patients. Emails were distributed once a week on a regular basis for short-term and once a month for long-term outcome and contained standardized text modules about the patients’ present clinical state. Every email included the following information: admission and discharge date, radiologic and clinical diagnosis, therapy, clinical condition at the time of admission and discharge and 90 days post-stroke, Modified Rankin Scale (mRS) pre-stroke, at admission, at discharge and 90 days post-stroke, time-window from onset of symptoms to hospital admission, time-window from onset of symptoms to reperfusion of the occluded vessel. Patients were questioned by telephone 90 to 110 days after their hospital admission for long-term evaluation. Treatment was performed as described previously [21].

### Survey participants

Initially, 49 of 49 employees participated in our survey. Ten of 49 (20%) participants dropped out because they were on maternity leave or were no longer employed in our department during the second survey round. Consequently, 39 of 49 total employees (80%) participated in both rounds of our survey. Most respondents (69%) were female. Of the 39 respondents, 16 (41%) were radiologists, 16 (41%) were radiographers, and 7 (18%) were researchers. All radiologists and radiographers in our department do diagnostic work (mainly computed tomography, magnet resonance tomography, and angiography) and all but two senior radiologists and two senior radiographers also do interventional work. The latter two radiologists and two technicians have a long experience in neuro-interventions and stroke-therapy. Workstations alternate regularly, often on a daily basis. Emergency physicians, neurologists, and anaesthesiologists have important functions during interventional stroke therapy, but are not employed by our department of Neuroradiology and are consequently not part of this survey.

### Patient cohort

Frequency distributions and descriptive statistics of the patient cohort results were as follows: there were 196 endovascularly treated stroke patients from September 2019 until June 2020, 50.5% of whom were women. Mean age was 73.5±13.5. Thrombectomy was successful (eTICI score 2b-3) in 87% of patients. In-hospital mortality was 28%. The average length of stay in the hospital was 13.7±12.0 days. In the long-term cohort, for whom 90 day follow up was available and which only included patients from September 2019 to February 2020 (n = 97), 35% of patients had a favorable outcome (mRS 0–2) and 65% had an unfavorable outcome (mRS 3–6), with a mortality rate of 38% (mRS 6) (S1 Table).

### Statistical analysis

Spearman-Rho correlation coefficients were calculated between pairs of variables. Mann-Whitney-U and Kolmogorov-Smirnov tests were used to compare differences between groups. Differences between repeated measurements were calculated using paired t-test and Wilcoxon-signed-rank test. P values under the α-level of 0.05 were defined as significant. All statistical analyses were performed with SPSS software version 26 (IBM, Armonk, New York, USA).

## Results

### Questionnaire results

The highest bivariate Spearman-Rho correlation coefficients found are between work satisfaction and work meaningfulness (r = 0.733, p <0.001), profession and wish for feedback (r = 0.608, p <0.001), work meaningfulness and valuation of EST (r = 0.582, p <0.001), work experience and valuation of EST (r = -0.558, p <0.001), work satisfaction and valuation of EST (r = 0.546, p <0.001) and work satisfaction and wage satisfaction (r = 0.534, p <0.001) (S2 Table).

**Fig 1:**
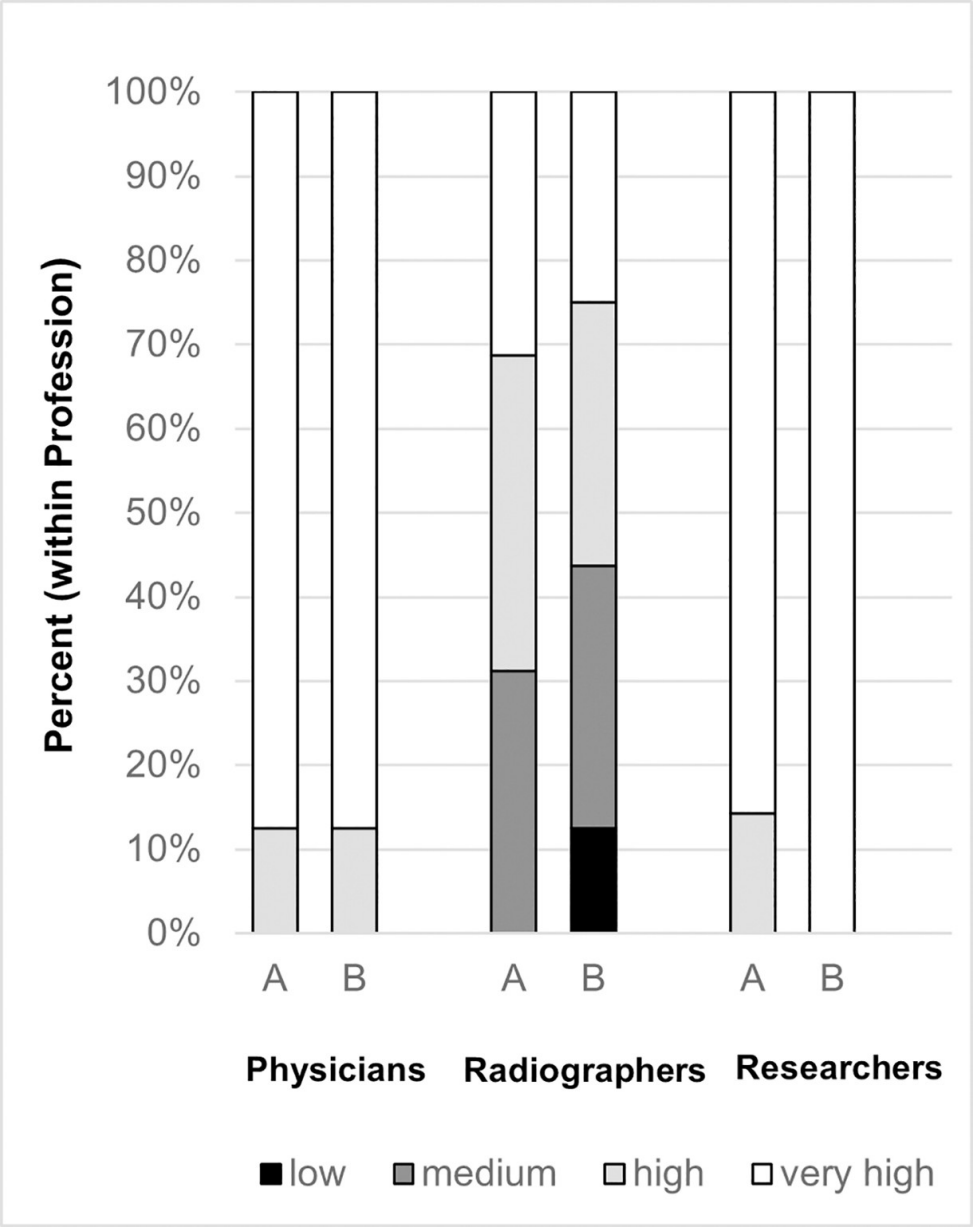
Valuation of usefulness of EST before (A) and after (B) the feedback-period.

Table 1 presents the frequency distributions of questionnaire responses from both rounds of the survey. Closer inspection of the Table shows several significant differences in responses to the study variables between the three analysed professions. In summary, in the first round of the survey, physicians were more satisfied with their work than radiographers (p = 0.034), physicians had a higher perceived work meaningfulness than researchers had (p = 0.045), both researchers and radiographers were significantly less satisfied with their salary compared to physicians (p = 0.028 and p = 0.001, respectively) and, as illustrated in Fig 1, radiographers valuated the usefulness of EST significantly lower than physicians and researchers did (p = 0.001 and p = 0.017, respectively). Additionally, researchers had the lowest desire for feedback about patients’ conditions compared to radiographers and physicians (p = 0.016 and p = 0.001, respectively), but even radiographers had significantly less desire for feedback than physicians (p = 0.015). No significant differences were found for work volition and subjective competence-assessment among the professions.

**Table 1:** Frequency of questionnaire variables in January and July 2020, separated by profession and in total, in %*(actual numbers reported in brackets).

Variable	Physicians (n = 16)		Radiographers (n = 16)		Researchers (n = 7)		Total (n = 39)	
	January	July	January	July	January	July	January	July
Work satisfaction								
• Low	0% (0)	0% (0)	6% (1)	6% (1)	0% (0)	0% (0)	3% (1)	3% (1)
• Medium	19% (3)	13% (2)	31% (5)	44% (7)	29% (2)	29% (2)	26% (10)	28% (11)
• High	81% (13)^a^	88% (14)	63% (10)^a^	50% (8)	71% (5)	71% (5)	72% (28)	69% (27)
Perceived work meaningfulness								
• Very low	6% (1)	6% (1)	0% (0)	0% (0)	0% (0)	0% (0)	3% (1)	3% (1)
• Low	6% (1)	13% (2)	19% (3)	44% (7)	0% (0)	29% (2)	10% (4)	28% (11)
• Medium	38% (6)	50% (8)	62% (10)	50% (8)	100% (7)	43% (3)	59% (23)	49% (19)
• High	50% (8)	31% (5)	19% (3) ^b^	6% (1)	0% (0) ^b^	29% (2)	28% (11)	21% (8)
Work Volition								
• Very low and low	19% (3)	-	0% (0)	-	14% (1)	-	10% (4)	-
• Medium	13% (2)	-	13% (2)	-	0% (0)	-	10% (4)	-
• High and very high	69% (11)	-	88% (14)	-	86% (6)	-	79% (31)	-
Wage satisfaction								
• Very low and low	19% (3)^c^	19% (3)	50% (8)^c^	63% (10)	43% (3)^c^	29% (2)	36% (14)	38% (15)
• Medium	6% (1)	31% (5)	31% (5)	25% (4)	14% (1)	43% (3)	18% (7)	31% (12)
• High and very high	75% (12)	50% (8)	19% (3)	13% (2)	43% (3)	29% (2)	46% (18)	31% (12)
Wish for patient feedback								
• Very low	0% (0)	6% (1)	0% (0)	38% (6)	0% (0)	0% (0)	0% (0)	18% (7)
• Low	0% (0)	0% (0)	0% (0)	6% (1)	0% (0)	0% (0)	0% (0)	3% (1)
• Medium	6% (1)	25% (4)	19% (3)	19% (3)	71% (5)	0% (0)	23% (9)	18% (7)
• High	31% (5)	19% (3)	63% (10)	31% (5)	29% (2)	43% (3)	44% (17)	28% (11)
• Very high	63% (10)^d^	50% (8)	19% (3)^d^	6% (1)	0% (0)^d^	57% (4)	33% (13)	33% (13)
Valuation of EST								
• Low	0% (0)	0% (0)	0% (0)	13% (2)	0% (0)	0% (0)	0% (0)	5% (2)
• Medium	0% (0)	0% (0)	31% (5)	31% (5)	0% (0)	0% (0)	12% (5)	13% (5)
• High	13% (2)	13% (2)	38% (6)	31% (5)	14% (1)	0% (0)	23% (9)	18% (7)
• Very high	88% (14)^e^	88% (14)	31% (5)^e^	25% (4)	86% (6) ^e^	100% (7)	64% (25)	64% (25)
Subjective competence assessment								
• Very low and low	13% (2)	13% (2)	6% (1)	0% (0)	14% (1)	14% (1)	10% (4)	8% (3)
• Medium	25% (4)	13% (2)	13% (2)	25% (4)	0% (0)	29% (2)	15% (6)	21% (8)
• High and very high	63% (10)	75% (12)	81% (13)	75% (12)	86% (6)	57% (4)	74% (29)	72% (28)

*Percentage rounded to the nearest whole number.

^a^ There was a significant difference in work satisfaction score between radiographers and physicians (p = 0.034)^1^.

^b^ There was a significant difference in work meaningfulness score between physicians and researchers (p = 0.045)^1^.

^c^ Radiographers are significantly less satisfied with their salary than physicians (p = 0.001)^1^. The same applies for researchers (p = 0.028)^2^.

^d^ The wish for patient feedback was significantly higher by physicians than by researchers (p = 0.001)^1^ and radiographers (p = 0.015)^2^. The wish for patient feedback was also significantly lower by researchers than by radiographers (p = 0.016)^2^.

^e^ Radiographers valuate IST less useful than physicians (p = 0.001)^1^ and researchers (p = 0.017)^2^.

^1^ The distributions differed between both groups, Kolmogorov-Smirnov p≤0.05.

^2^ The distributions were the same in both groups, Kolmogorov-Smirnov p≥0.05.

Abbreviations: Endovascular stroke therapy = EST.

Table 1 also provides some tendencies regarding changes in the second questionnaire: perceived work-meaningfulness tends to have decreased in all professions. Radiographers tend to value EST less useful than before receiving feedback (Fig 1). Wage satisfaction tends to have decreased among radiographers and physicians. The Wilcoxon-signed-rank test reveal significant differences concerning the wish for patient feedback. It has significantly decreased among radiographers (p = 0.007), while it has, on the contrary, significantly increased among researchers (p = 0.034). The remaining variables analysed in the comparative tests were not significant (S3 Table).

Table 2 presents the results of the additional questions from round 2 of the survey.

**Table 2:** Frequency of questionnaire variables in July, separated by profession and in total, in %* (actual numbers reported in brackets).

Variable	Physicians (n = 16)	Radiographers (n = 16)	Researchers (n = 7)	Total (n = 39)
Training needs				
• Very low and low	6% (1)	6% (1)	0% (0)	5% (2)
• Medium	19% (3)	38% (6)	43% (3)	31% (12)
• High and very high	75% (12)	56% (9)	57% (4)	64% (25)
Motivating Factors are				
• Good clinical outcome (mRS ≤ 2)	100% (16)	94% (15)	100% (7)	97% (38)
• Good interventional outcome (eTICI >2a)	63% (10)^a^	25% (4)^a^	0% (0)	36% (14)
• Good outcome in general	63% (10)^b^	13% (2)^b^	0% (0) ^b^	31% (12)
• Significant improvement of mRS	25% (4)	50% (8)	43% (3)	39% (15)
• Others	0% (0)	6% (1)	0% (0)	3% (1)
Demotivating factors are				
• Unfavorable clinical outcome (mRS >2)	63% (10)	88% (14)	86% (6)	77% (30)
• Unfavorable interventional outcome (eTICI <2b)	38% (6)	44% (7)	14% (1)	36% (14)
• Death as an outcome (mRS = 6)	70% (11)	75% (12)	86% (6)	74% (29)
• Bad outcome in general	31% (5)	25% (4)	14% (1)	26% (10)
• Others	6% (1)	6% (1)	0% (0)	5% (2)
Patient cases that particularly affected were				
• Young patients	75% (12)	88% (14)	86% (6)	82% (32)
• Patients in the same age as oneself	25% (4)^c^	56% (9)	71% (5)^c^	46% (18)
• Patients in the same age as ones’ parents	19% (3)	44% (7)	14% (1)	28% (11)
• Patients without any risk factors	19% (3)	31% (5)	57% (4)	31% (12)
• Patients with unfavorable clinical outcome though good initial conditions**	69% (11)	56% (9)	57% (4)	62% (24)
Estimation of stroke-disease severity				
• More severe than before	25% (4)^d^	75% (12)^d^	57% (4)	51% (20)
• Less severe than before	13% (2)	6% (1)	0% (0)	8% (3)
• No change in estimation	63% (10)	19% (3)	43% (3)	41% (16)
Patient cases that were remembered the most				
• Bad outcome cases	81% (13)	75% (12)	57% (4)	74% (29)
• Good outcome cases	44% (7)	19% (3)	14% (1)	28% (11)
• Both cases	31% (5)	19% (3)	14% (1)	23% (9)
• Young patient cases	50% (8)	69% (11)	71% (5)	62% (24)
• Patient without risk factors cases	6% (1)	25% (4)	29% (2)	18% (7)
Degree of influence on work satisfaction through feedback				
• Very low and low	6% (1)	0% (0)	14% (1)	5% (2)
• Medium	25% (4)^e^	38% (6)^e^	71% (5)^e^	39% (15)
• High and very high	69% (11)	63% (10)	14% (1)	56% (22)
The systematic feedback				
• Is educational	75% (12)^f^	38% (6) ^f^	100% (7)	64% (25)
• Motivates for improving skills	56% (9)^g^	13% (2)^g^	14% (1)	31% (12)
• Demotivates	13% (2)	38% (6)	0% (0)	21% (8)
• Makes me doubt the usefulness of EST	19% (3)^h^	56% (9)^h^	0% (0)^h^	31% (12)
• Confirms my assessment of EST as useful	44% (7)	31% (5)	29% (2)	36% (14)
• Has no influence on my work	19% (3)	25% (4)	43% (3)	26% (10)
• Increases the personal appreciation of my work	50% (8)	19% (3)	29% (2)	33% (13)

* Percentages are rounded to the nearest whole number.

** Though good time-window and good interventional outcome.

^a^ Physicians consider a good interventional outcome significantly more often as a motivating factor than researchers (p = 0.007)^1^ and radiographers (p = 0.035)^2^.

^b^ Physicians consider a good clinical outcome in general significantly more often as a motivating factor than researchers (p = 0.007)^1^ and radiographers (p = 0.004)^1^.

^c^ Physicians are significantly less affected by patients in the same age as oneself than researchers (p = 0.04)^2^.

^d^ Radiographers estimate stroke significantly more often as more severe after feedback was given than physicians (p = 0.009)^1^. Spearman Rho correlation between profession and estimation of stroke-severity was significant (r = 0.361, p = 0.024).

^e^ Researchers’ work satisfaction is significantly less influenced through patients’ feedback than the work satisfaction of radiographers (p = 0.019)^2^ and physicians (p = 0.025)^2^.

^f^ Radiographers consider feedback significantly less often as educational than physicians (p = 0.035)^2^ and researchers (p = 0.007)^1^.

^g^ Radiographers consider feedback significantly less often as motivating than physicians (p = 0.01)^2^. Spearman Rho correlation between profession and considering feedback as motivating was significant (r = 0.419, p = 0.008).

^h^ Radiographers significantly more often agree to the fact that the feedback makes them doubt the usefulness of EST than physicians(p = 0.031)^2^ and researchers (p = 0.013)^2^.

^1^ The distributions differed between both groups, Kolmogorov-Smirnov p≤0.05.

^2^ The distributions were the same in both groups, Kolmogorov-Smirnov p≥0.05.

Abbreviations: Endovascular stroke therapy = EST.

In general, more than half of the participants (56%) believe that receiving feedback has a high or very high influence on their work satisfaction. 64% considered the feedback educational. Most of the participants (74%) primarily remembered patients with unfavorable outcomes. Less than one third of participants (28%) stated that they also remember patients with good outcome. Almost everyone (97%) agreed that the most motivating factor is a good clinical outcome. 77% said that the most demotivating factor is an unfavorable clinical outcome and 74% stated that especially death as the worst outcome is the most demotivating.

**Fig 2:**
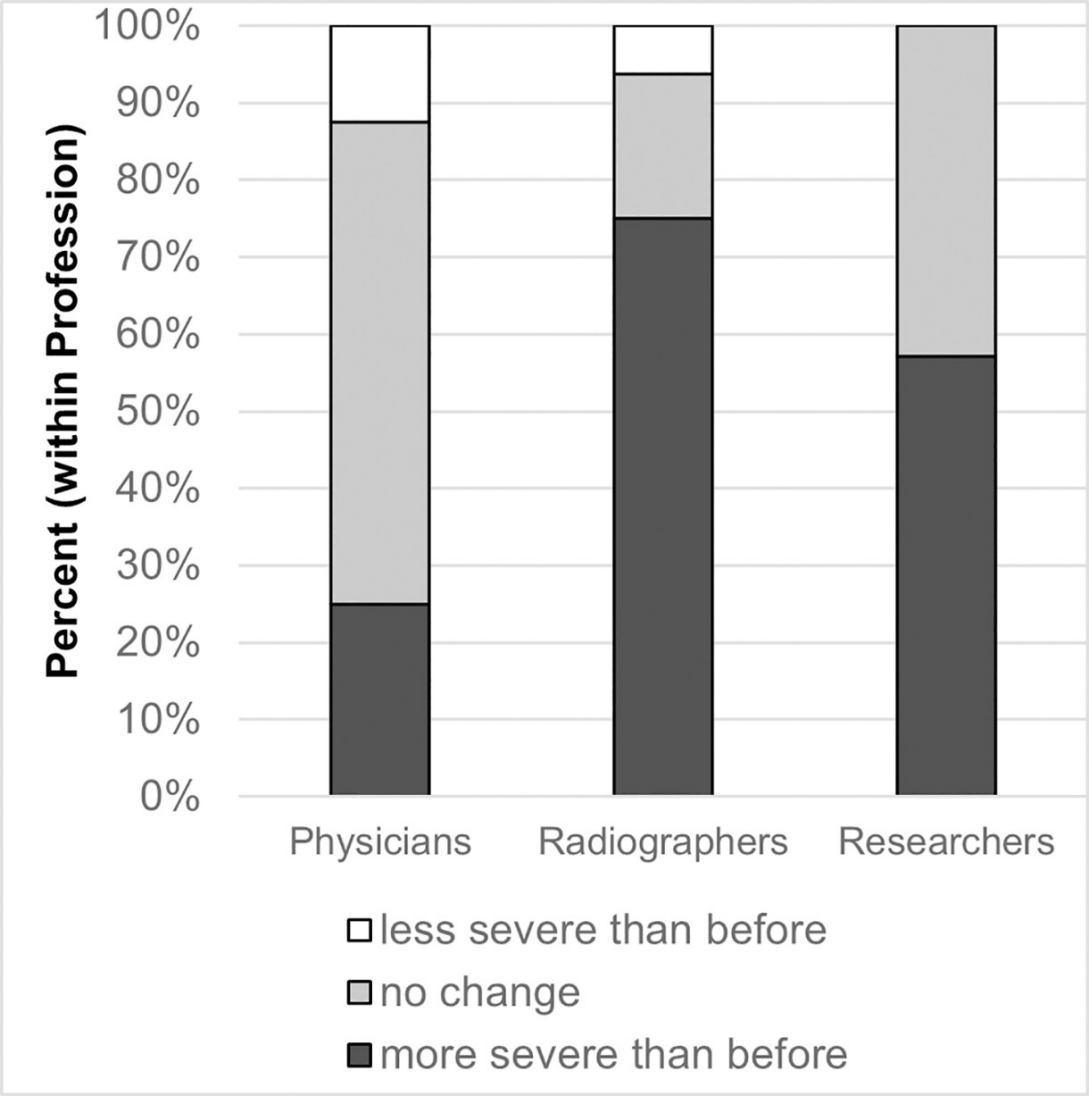
Estimation of stroke-severity after the feedback-period.

Statistical analysis revealed that researchers and radiographers count a good interventional outcome less often as a motivating factor than physicians did (p = 0.007 and p = 0.035, respectively). They also less often state that a good interventional outcome is just as motivating as a good clinical outcome (p = 0.007 and p = 0.004, respectively). Contrary to this, an unfavorable interventional outcome was equally rated as demotivating by radiographers as it was by physicians (44% and 38%, p = 0.276). As shown in Fig 2, 75% of radiographers said that they estimate stroke as more severe than before receiving feedback, while only 25% of physicians stated the same (p = 0.009). There was a significant positive correlation between profession and estimation of stroke-severity (r = 0.361, p = 0.024). Altogether half of the participants (51%) estimated stroke as more severe than before receiving feedback. While radiographers and physicians are equally influenced by the feedback, researchers cite a smaller influence on their work satisfaction compared to radiographers and physicians (p = 0.019 and p = 0.025, respectively). No significant differences in training needs and demotivating factors were found between the professions.

Researchers and physicians both considered the feedback more often as educational than radiographers did (p = 0.007 and p = 0.035, respectively). Physicians considered feedback more often as motivating than radiographers did (p = 0.01). Radiographers more often considered the feedback as making them doubt the usefulness of EST than physicians and researchers did (p = 0.031 and p = 0.013, respectively).

## Discussion

### Job satisfaction after feedback

Contrary to our expectations, this study did not find a significant increase in work satisfaction and meaningfulness among employees after they had received systematic feedback about their patients’ conditions. Most of the literature would consider the described increasing isolation and separation of radiology staff since the introduction of PACS and electronical medical records as threatening for job satisfaction [22,23]. The detachment of physicians and radiographers from the further course of the patient after initial emergency-treatment is hypothesized to result in employees questioning the value and success of their work, not being able to experience the potentially significant improvement of their patients’ health status. Research claims that radiology staff cannot experience achievement and competence at work as easily as other specialties can and they do not encounter gratitude from the patients for their work [22]. This sense of ineffectiveness at work (low personal accomplishment) is one of the well-known burnout components, indicating a possible relation between the separation of radiology staff and the higher burnout frequencies found in radiologists compared to other medical specialties [24,25]. According to these assumptions we anticipated an increase in work satisfaction through systematic feedback, but we could not provide scientific evidence that less isolation and more information about their patients’ health outcomes led to an increased work satisfaction or meaningfulness among our neuroradiology staff.

We also cannot confirm the results of the study by Friedberg et. al, in which receiving helpful quality performance data correlated with higher work satisfaction [14]. In fact, radiographers considered the feedback less often educational and their desire for feedback was significantly lower compared to physicians and even decreased further after having received feedback. Especially considering the fact that we only reported back information about stroke patients, it seems equally logical that awareness of the patients’ outcome can also have a demotivating effect, since 40–67.4% of large vessel occlusion stroke interventions have an unfavorable outcome [26–31].

### Assessment of the usefulness of EST and stroke-severity

One of the most interesting findings was the disparity between radiographers and physicians regarding their estimation of usefulness of EST and stroke-severity. Radiographers valuated the usefulness of EST the least of all, and they rated EST even less useful after the feedback period, while physicians rated it consistently as highly or very highly useful. A possible explanation for this discrepancy might be different expectations in therapy effects. Although we did not specifically examine personal expectations in our study, our results suggest that the disappointment over poor clinical patient outcomes affected the desire for feedback to such an extent. In accordance with this assumption, three-quarter of participants stated that they primarily remembered patients with unfavorable outcomes. While our favorable outcome rate of 35% is in the range of the expected, the mortality rate of 38% during the time frame of our analysis is higher than our usual average, clearly higher than the 9–19% reported in the randomized trials and also higher than the 29% reported in the German stroke registry [32–34]. When put into clinical perspective, these differences are mainly explained by the real life setting with less strict inclusion criteria, with our cohort including multimorbid patients with high pre-stroke mRS, ELVO in the posterior circulation, very low ASPECT scores, and prolonged and unknown time-windows. In fact, mortality in such patients with rather unfavorable initial conditions is reported to be in ranges around 41–45% [35]. Given that untreated ELVO has a mortality of approximately 80%, any reduction of mortality should appear worthwhile [36]. However, the inherently poor prognosis of many patients, who may not have been treated previously, is likely to clash with the expectation of a good outcome. This is understandable given that overall stroke-associated mortality rates continued to decline over the last few decades from 117.25/100.000/year in the pre-thrombolysis era (1990) to 88.41/100.000/year in 2010 [37]. It is expected that mortality decreases even further since mechanical thrombectomy has been established as standard of care in 2015, especially as mechanical thrombectomy addresses ELVO, which has a particularly high mortality. We hypothesize that with the development of new therapy options and the continuously declining mortality of stroke patients, medical professionals developed a trivialized perception of the disease. This is expressed in the unrealistic assessment of the stroke-severity by radiographers in our department, of whom three-quarter confirmed that they estimated stroke as more severe than before receiving feedback, while only one quarter of physicians confirmed the same.

### Different motivational factors

Different motivational factors also support the hypothesis that false expectations prevail: Only one quarter of radiographers considered a good interventional outcome as motivating. For physicians, on the other hand, a good interventional outcome often was equally motivating as a good clinical outcome. This result suggests that a good interventional outcome for physicians represents having done their job properly and that the further clinical course is not fully in their power. Actually, only approximately half of the patients with a good interventional outcome have a favorable clinical outcome [38]. Since the feedback had no negative impact on job satisfaction and the assessment of mechanical thrombectomy for physicians despite the unfavorable outcomes, we assume that physicians accept a certain detachment between procedural and clinical outcome, whereas radiographers do not do so to the same extent.

### Valuation of therapy correlates with satisfaction

A further important finding was that work satisfaction and perception of mechanical thrombectomy are associated: Our correlation analyses indicate that employees with a high work meaningfulness and high work satisfaction both rate EST as more useful and vice versa. At the same time, less satisfied employees and employees with a lower sense of work meaningfulness rate EST as less useful. Although it is not fully clear whether low work satisfaction results in low perception of mechanical thrombectomy or vice-versa, this finding may help to identify employees that are dissatisfied with their job and therefore at risk for long-term effects such as burn-out.

### Researchers appreciate the feedback

One unanticipated finding was that researchers, who had the lowest desire for feedback in the beginning, showed the highest desire for feedback of all professions after the feedback period. These results are likely due to the fact that all researchers considered the feedback as educational and no researcher experienced it as demotivating.

## Limitations

Our relatively small sample size is a limitation, which was defined by the fixed number of permanent staff in the participating neuroradiology department. However, as our department is relatively large compared to many other hospitals, our sample size is rather representative and should not be assessed as exceptionally small in this relation. Also, our survey response rates were relatively high, assuming that the results of this study are representative for the neuroradiology staff in our department. The risk of a selection or a non-response bias is low, because all employees participated in our survey and the 10 non-participants during the second survey round were either on maternity leave or no longer employed in our department.

Moreover, our study is rather exploratory and descriptive and as such it is unlikely that our findings are fully transferable to other medical centres. Also, given the exploratory nature of our study, we decided not to conduct complex multivariable analyses, especially given the small sample size and the large number of variables. In addition, some potential confounding variables could not be evaluated. For example, workload, quality of equipment, training opportunities, work climate and work organisation have been identified in a previous study as possible influential factors for work satisfaction [39]. On account of these issues, our results are best interpreted as associations rather than as irrevocable proof of causality. Nevertheless, our study gives valuable conclusions for hospitals, that have not yet established systematic clinical feedback. It proofs that a survey as such is worthwhile because it provides insight into the thought processes and expectations of employees and thereby gives first approaches to detect factors than can contribute to an enhanced work satisfaction among the staff.

## Conclusion

We expected the work satisfaction and work meaningfulness to increase, due to making it possible for employees to witness the clinical course of their patients. However, work satisfaction and work meaningfulness did not change after receiving feedback for six months. In fact, the desire for feedback decreased in radiographers.

Key findings were that it was rather unfavorable outcomes that affected work satisfaction of medical staff and that less satisfied employees and employees with a low work meaningfulness rated EST as less useful. We were also able to identify the unexpected issue that radiographers in particular had an unrealistic perception of stroke-severity and the potentially beneficial effects of mechanical thrombectomy.

Taken together, the findings of our study indicate that systematic clinical feedback via email is advantageous, as it is educative for the staff and it can help to assess the severity of a disease and the therapeutic effects more realistically. Also, even though our results may not be transferable to all other hospitals, our study suggests that evaluating work satisfaction and the perception of treatment may help to identify unexpected issues and may therefore help to find specific measures that increase work satisfaction and motivation.

## Supporting information

S1 Table Statistical analysis of the patient cohort September 2019-June 2020 (n = 196). (DOCX)

S2 Table Descriptive statistics and factor correlations (Spearman Rho) of study variables. (DOCX)

S3 Table Results of the pre- to postintervention comparative test. (DOCX)

## Abbreviations

1.ELVO: Emergent large vessel occlusion
2. mRS: Modified rankin scale
3. EST: Endovascular stroke therapy
