# Supplementary material for: Work satisfaction among neuroradiology staff after receiving follow up reports of thrombectomy stroke patients
Source: PLoS One. 2021 May 19;16(5):e0251889. doi: 10.1371/journal.pone.0251889 (PMC8133452; doi:10.1371/journal.pone.0251889)
Supplement: S1 Table — (DOCX) [file pone.0251889.s001.docx]

| Variable | Count (*mean;**median) | Percent (*SD;**IQR) |
| --- | --- | --- |
| Sex   - Female - Male | 99  97 | 50.5 %  49.5 % |
| Age (years) | 73.52* | 13.53* |
| Clinical Syndrome   - Anterior: Carotid flow area - Posterior: Vertebral or basilar cerebral artery | 179  17 | 91.3 %  8.7 % |
| Stenting   - No stent - Stent after thrombectomy - Stent before thrombectomy - Thromboendarterectomy - Balloon dilatation - Intracranial stent - Stent only | 144  28  2  18  2  1  1 | 73.5 %  14.3 %  1.0 %  9.2 %  1.0 %  0.5 %  0.5 % |
| Interventional method   - Thrombectomy - Intravenous lysis and thrombectomy - Intravenous and intra-arterial lysis - Intra-arterial lysis - Intra-arterial lysis and thrombectomy - Intra -venous and -arterial lysis and thrombectomy - Balloon percutaneous transluminal angioplasty | 104  80  3  1  5  2  1 | 53.1%  40.8%  1.5%  0.5%  2.6%  1.0%  0.5% |
| TICI score post-thrombectomy (n=192)   - 0 - 1 - 2a - 2b - 2c - 3 | 18  3  4  39  21  107 | 9.4%  1.6%  2.1%  20.3%  10.9%  55.7% |
| NIHSS at admission (n=173) | 14** | 11** |
| MRS score pre-stroke (n=178)   - 0 - 1 - 2 - 3 - 4 - 5 | 117  16  14  19  11  1 | 65.7%  9.0%  7.9%  10.7%  6.2%  0.6% |
| MRS score at dismissal (N=196)   - 0 - 1 - 2 - 3 - 4 - 5 - 6 | 10  15  31  35  33  18  54 | 5.1%  7.7%  15.8%  17.9%  16.8%  9.2%  27.6% |
| MRS score 90 days post-stroke (N=97)   - 0 - 1 - 2 - 3 - 4 - 5 - 6 | 12  11  11  12  8  6  37 | 12.4%  11.3%  11.3%  12.4%  8.2%  6.2%  38.1% |
| Alteration of MRS score post- compared to pre-stroke (N=94)   - 0 - 1 - 2 - 3 - 4 - 5 - 6 | 19  13  12  18  12  5  15 | 20.2%  13.8%  12.8%  19.1%  12.8%  5.3%  16.0% |
| Death cause (N=65)   - Cerebrovascular - Pneumonia/ Sepsis - Cardial death - Other cause - Unknown cause | 30  14  3  5  13 | 46.2%  21.5%  4.6%  7.7%  20.0% |
| Symptom-onset to admission (hours) | 1.73* | 7.35* |
| Symptom-onset to reperfusion (hours) | 4.05* | 7.93* |

**S1 Table.** Statistical analysis of the patient cohort September 2019-June 2020 (n=196)
