# Supplementary material for: Work satisfaction among neuroradiology staff after receiving follow up reports of thrombectomy stroke patients
Source: PLoS One. 2021 May 19;16(5):e0251889. doi: 10.1371/journal.pone.0251889 (PMC8133452; doi:10.1371/journal.pone.0251889)
Supplement: S2 Table — (DOCX) [file pone.0251889.s002.docx]

|  | T1 | 2 | 3 | 4 | 5 | 6 | 7 | 8 | 9 |
| --- | --- | --- | --- | --- | --- | --- | --- | --- | --- |
| 1. Profession  2. Work experience (y)  3. Wage satisfaction  4. Independent job choice  5. Wish for feedback  6. Valuation of therapy  7. Competence-assessment  8. Meaningful work  9. Work satisfaction  Mean  SD | -  0.3  0.487**  -0.348*  0.608**  0.226  -0.112  0.326*  0.186 | -  -0.240  0.042  -0.113  -0.558**  0.056  -0.244  -0.337*  7.15  7.89 | -  -0.127  0.340*  0.398*  -0.140  0.388*  0.534** | -  -0.004  -0.031  -0.017  0.388*  0.534* | -  0.267  0.340*  0.381*  0.215 | -  -0.022  0.582**  0.546** | -  0.119  0.162 | -  0.733**  36.18  6.76 | -  19.9  3.48 |

**S2 Table.** Descriptive statistics and factor correlations (Spearman Rho) of study variables. *p = < 0.05, ** p = < 0.01
