# Supplementary material for: Work satisfaction among neuroradiology staff after receiving follow up reports of thrombectomy stroke patients
Source: PLoS One. 2021 May 19;16(5):e0251889. doi: 10.1371/journal.pone.0251889 (PMC8133452; doi:10.1371/journal.pone.0251889)
Supplement: S3 Table — (DOCX) [file pone.0251889.s003.docx]

| Variable | Test used | Outliers | Shapiro-Wilk p | Test statistic | P-Value | Pre-; Post-Mean |
| --- | --- | --- | --- | --- | --- | --- |
| Work Satisfaction | Paired t-test | none | p = 0.03 | t(38) = 1.013 | 0.318 | 19.9; 19.44 |
| Work Meaningfulness | Paired t-test | none | p = 0.038 | t(38) = 1.374 | 0.178 | 36.18; 34.92 |
| Valuation of interventional stroke therapy   - In general - From Physicians - From Radiographers - From Scientists | Wilcoxon signed ranked test  Wilcoxon signed ranked test  Wilcoxon signed ranked test  Wilcoxon signed ranked test | -  -  -  - | -  -  -  - | z =-1.027  z =0.0  z =-1.406  z =-1.0 | 0.305  1.0  0.16  0.317 | 4.51; 4.41  4.88; 4.88  4.0; 3.69  4.86; 5.0 |
| Subjective competence assessment   - In general - From Physicians - From Radiographers - From Scientists | Wilcoxon signed ranked test  Wilcoxon signed ranked test  Wilcoxon signed ranked test  Wilcoxon signed ranked test | -  -  -  - | -  -  -  - | z =-0.046  z =0.707  z =0.0  z =-0.557 | 0.963  0.48  1.0  0.577 | 3.79; 3.74  3.69; 3.69  3.94; 3.94  3.71; 3.43 |
| Desire for feedback about patients’ conditions   - In general - From Physicians - From Radiographers - From Scientists | Wilcoxon signed ranked test  Wilcoxon signed ranked test  Wilcoxon signed ranked test  Wilcoxon signed ranked test | -  -  -  - | -  -  -  - | z =-2.127  z =-1.725  z =-2.699  z =-2.121 | 0.033  0.084  0.007  0.034 | 4.1; 3.56  4.56; 4.06  4.0; 2.63  3.29; 4.57 |

**S3 Table.** Results of the pre- to postintervention tests
