# Supplementary material for: Work satisfaction among neuroradiology staff after receiving follow up reports of thrombectomy stroke patients
Source: PLoS One. 2021 May 19;16(5):e0251889. doi: 10.1371/journal.pone.0251889 (PMC8133452; doi:10.1371/journal.pone.0251889)
Supplement: Attachment — Submitted filename: Response to Reviewers.docx [file pone.0251889.s004.docx]

Dear Editor,

Dear reviewers,

We would like to thank you for the thoughtful and favorable reviews and the opportunity to revise our manuscript. We believe that our manuscript has benefitted considerably from the remarks and we hope that we addressed the mentioned issues to your satisfaction. Please also note that we made some minor changes in the text and minor changes in design, the latter to adapt to the journal style.

Below you can find our responses to the reviewer’s comments. Please also find attached our revised manuscript in two versions: with and without tracked changes.

With our highest regards,

The authors

Comments to the Authors

**REVIEWER 1:**

1. Abstract: nothing to add.
2. Introduction:
   Specifying the difficulties of implementing clinical-radiologic rounds would be recommendable, especially considering these precise difficulties are the ones motivating the e-mail centric design of the study

**Response:**

**We thank the reviewer for this recommendation and modified the text accordingly:**

*However, clinical-radiologic rounds are often difficult to implement in the existing hospital structure,* ***as rounds are often time-consuming and time schedules of staff can vary from day to day*** *[6, 13].*

3. Methods
   Even if mentioned later in the section of Results, study population should be described in this section as well.

**Response:**

**The paragraphs about participants and patients are now found in the Methods section.**

4. Results
   It is stated that 39 of 49 total employees available participated in both rounds of the survey, however it is not stated if that 80% signify that there were 10 employees that dropped out between rounds of the survey, or if there were 10 employees who declined to take part of the study at all. If there was a 20% drop out between rounds, was there any registration for the reasons behind it?
   Even though short-term feedback is defined from admission to discharge, no mention is made about of how long that time is, or which was the mean hospital stay of the patients included in the sample.

**Response:**

**The reviewer rightfully points out that it does not become clear, why and when the ten non-participants dropped out of the study. Initially all employees took part. Ten participants from the first round of the survey could no longer take part in the second round of the survey because they were no longer employed in the department or were on maternity leave. This is now specified in the Methods section:**

*“****Initially, 49 of 49 employees participated in our survey. Ten of 49 (20%) participants dropped out because they were on maternity leave or were no longer employed in our department during the second survey round.“***

**The reviewer also refers to the missing information about the duration of hospital stay of patients. We added this information in the Methods section:**

*“****The average length of stay in the hospital was 13.7±12.0 days.”***

5. Discussion and Limitations
   No mention is made from the possible selection bias from the voluntary participation in which the study design rests.

**Response**:

**The reviewer addresses the important issue of a selection bias. While we only took up the issue of a non-response bias in our limitations section, we now added the issue of a selection bias:**

*“The risk of a* ***selection*** *or a non-response* ***bias is low****,* ***because all employees participated in our survey and the 10 non-participants during the second survey round were either on maternity leave or no longer employed in our department****.”*

**REVIEWER 2:**

1. The results are interesting, as it seems to make a difference between the training of physicians, radiographers and researchers, however, I would like to clarify who is part of each group, for example, in the group of physicians, are emergency physicians and neurologists included?, in the group of radiologists, are diagnostic radiologists and interventional neuroradiologists included?, etc.

**Response:**

**We thank the reviewer for pointing out this inaccuracy. All radiologists and radiographers in our department do diagnostic work and all but two senior radiologists and two senior radiographers also do interventional work. The latter two radiologists and two technicians have a long experience in neuro-interventions and stroke-therapy. Workstations alternate regularly, often on a daily basis. Emergency physicians, neurologists, and anaesthesiologists are not employed by our department and were therefore not part of this survey.**

*“****All radiologists and radiographers in our department do diagnostic work (mainly computed tomography, magnet resonance tomography, and angiography) and all but two senior radiologists and two senior radiographers also do interventional work. The latter two radiologists and two technicians have a long experience in neuro-interventions and stroke-therapy. Workstations alternate regularly, often on a daily basis. Emergency physicians, neurologists, and anaesthesiologists have important functions during interventional stroke therapy, but are not employed by our department of Neuroradiology and are consequently not part of this survey.”***

2. Could you say if scientists and researchers mean the same thing in your writing?

**Response:**

**Using “scientists” and “researchers” was an unnecessarily awkward inacurracy. We now consistently refer to “researchers”.**

3. Could you define the meaning of PACS? This way anyone who reads your article will know what you mean.

**Response:**

**We thank the reviewer for pointing out this inaccuracy. We now spelled out this abbreviation** **in the text: *Picture Archiving and Communication System (PACS).***
