# Supplementary material for: Work satisfaction among neuroradiology staff after receiving follow up reports of thrombectomy stroke patients
Source: PLoS One. 2021 May 19;16(5):e0251889. doi: 10.1371/journal.pone.0251889 (PMC8133452; doi:10.1371/journal.pone.0251889)
Supplement: Peer review history

# Peer review: Decision Letter 0

**DOI:** 10.1371/journal.pone.0251889.r001

**Authors:** Miguel A. Barboza

25 Feb 2021

PONE-D-21-03388

Work satisfaction among neuroradiology staff after receiving follow up reports of thrombectomy stroke patients

PLOS ONE

Dear Dr. Nikoubashman,

Thank you for submitting your manuscript to PLOS ONE. After careful consideration, we feel that it has merit but does not fully meet PLOS ONE’s publication criteria as it currently stands. Therefore, we invite you to submit a revised version of the manuscript that addresses the points raised during the review process.

Please take a close look to reviewers' suggestions, mainly in terms of clarifying methodological issues and definitions related to the survey. 

Please submit your revised manuscript by Apr 11 2021 11:59PM. If you will need more time than this to complete your revisions, please reply to this message or contact the journal office at plosone@plos.org. When you're ready to submit your revision, log on to https://www.editorialmanager.com/pone/ and select the 'Submissions Needing Revision' folder to locate your manuscript file.

Please include the following items when submitting your revised manuscript:

- A rebuttal letter that responds to each point raised by the academic editor and reviewer(s). You should upload this letter as a separate file labeled 'Response to Reviewers'.
- A marked-up copy of your manuscript that highlights changes made to the original version. You should upload this as a separate file labeled 'Revised Manuscript with Track Changes'.
- An unmarked version of your revised paper without tracked changes. You should upload this as a separate file labeled 'Manuscript'.

If you would like to make changes to your financial disclosure, please include your updated statement in your cover letter. Guidelines for resubmitting your figure files are available below the reviewer comments at the end of this letter.

If applicable, we recommend that you deposit your laboratory protocols in protocols.io to enhance the reproducibility of your results. Protocols.io assigns your protocol its own identifier (DOI) so that it can be cited independently in the future. For instructions see: http://journals.plos.org/plosone/s/submission-guidelines#loc-laboratory-protocols

We look forward to receiving your revised manuscript.

Kind regards,

Miguel A. Barboza, MD, MSc

Academic Editor

PLOS ONE

Journal Requirements:

Please review your reference list to ensure that it is complete and correct. If you have cited papers that have been retracted, please include the rationale for doing so in the manuscript text, or remove these references and replace them with relevant current references. Any changes to the reference list should be mentioned in the rebuttal letter that accompanies your revised manuscript. If you need to cite a retracted article, indicate the article’s retracted status in the References list and also include a citation and full reference for the retraction notice.

When submitting your revision, we need you to address these additional requirements.

1. Please ensure that your manuscript meets PLOS ONE's style requirements, including those for file naming. The PLOS ONE style templates can be found at

https://journals.plos.org/plosone/s/file?id=wjVg/PLOSOne_formatting_sample_main_body.pdf and

https://journals.plos.org/plosone/s/file?id=ba62/PLOSOne_formatting_sample_title_authors_affiliations.pdf

2. PLOS requires an ORCID iD for the corresponding author in Editorial Manager on papers submitted after December 6th, 2016. Please ensure that you have an ORCID iD and that it is validated in Editorial Manager. To do this, go to ‘Update my Information’ (in the upper left-hand corner of the main menu), and click on the Fetch/Validate link next to the ORCID field. This will take you to the ORCID site and allow you to create a new iD or authenticate a pre-existing iD in Editorial Manager. Please see the following video for instructions on linking an ORCID iD to your Editorial Manager account: https://www.youtube.com/watch?v=_xcclfuvtxQ

3. Please amend your list of authors on the manuscript to ensure that each author is linked to an affiliation. Authors’ affiliations should reflect the institution where the work was done (if authors moved subsequently, you can also list the new affiliation stating “current affiliation:….” as necessary).

[Note: HTML markup is below. Please do not edit.]

Reviewers' comments:

Reviewer's Responses to Questions

**Comments to the Author**

1. Is the manuscript technically sound, and do the data support the conclusions?

The manuscript must describe a technically sound piece of scientific research with data that supports the conclusions. Experiments must have been conducted rigorously, with appropriate controls, replication, and sample sizes. The conclusions must be drawn appropriately based on the data presented. 

Reviewer #1: Yes

Reviewer #2: Yes

**********

2. Has the statistical analysis been performed appropriately and rigorously? 

Reviewer #1: N/A

Reviewer #2: Yes

**********

3. Have the authors made all data underlying the findings in their manuscript fully available?

The PLOS Data policy requires authors to make all data underlying the findings described in their manuscript fully available without restriction, with rare exception (please refer to the Data Availability Statement in the manuscript PDF file). The data should be provided as part of the manuscript or its supporting information, or deposited to a public repository. For example, in addition to summary statistics, the data points behind means, medians and variance measures should be available. If there are restrictions on publicly sharing data—e.g. participant privacy or use of data from a third party—those must be specified.

Reviewer #1: Yes

Reviewer #2: Yes

**********

4. Is the manuscript presented in an intelligible fashion and written in standard English?

PLOS ONE does not copyedit accepted manuscripts, so the language in submitted articles must be clear, correct, and unambiguous. Any typographical or grammatical errors should be corrected at revision, so please note any specific errors here.

Reviewer #1: Yes

Reviewer #2: Yes

**********

5. Review Comments to the Author

Please use the space provided to explain your answers to the questions above. You may also include additional comments for the author, including concerns about dual publication, research ethics, or publication ethics. (Please upload your review as an attachment if it exceeds 20,000 characters)

Reviewer #1: Had the pleasure to read and review the manuscript: “Work satisfaction among neuroradiology staff after receiving follow up reports of thrombectomy stroke patients”, an article analyzing the hypothetical positive impact of systematic feedback on work meaningfulness and work satisfaction following endovascular stroke treatment in neuroradiological staff. Before considering this manuscript for publication, I have some comments to add:

1) Abstract:

Nothing to add

2) Introduction:

Specifying the difficulties of implementing clinical-radiologic rounds would be recommendable, especially considering these precise difficulties are the ones motivating the e-mail centric design of the study

3) Methods

Even if mentioned later in the section of Results, study population should be described in this section as well.

4) Results

It is stated that 39 of 49 total employees available participated in both rounds of the survey, however it is not stated if that 80% signify that there were 10 employees that dropped out between rounds of the survey, or if there were 10 employees who declined to take part of the study at all. If there was a 20% drop out between rounds, was there any registration for the reasons behind it?

Even though short-term feedback is defined from admission to discharge, no mention is made about of how long that time is, or which was the mean hospital stay of the patients included in the sample.

5) Discussion and Limitations

No mention is made from the possible selection bias from the voluntary participation in which the study design rests.

6) Conclusions

Nothing to add

Reviewer #2: 1. The results are interesting, as it seems to make a difference between the training of physicians, radiographers and researchers, however, I would like to clarify who is part of each group, for example, in the group of physicians, are emergency physicians and neurologists included?, in the group of radiologists, are diagnostic radiologists and interventional neuroradiologists included?, etc.

2. Could you say if scientists and researchers mean the same thing in your writing?

3. Could you define the meaning of PACS? This way anyone who reads your article will know what you mean.

**********

6. PLOS authors have the option to publish the peer review history of their article (what does this mean?). If published, this will include your full peer review and any attached files.

If you choose “no”, your identity will remain anonymous but your review may still be made public.

**Do you want your identity to be public for this peer review?** For information about this choice, including consent withdrawal, please see our Privacy Policy.

Reviewer #1: No

Reviewer #2: No

[NOTE: If reviewer comments were submitted as an attachment file, they will be attached to this email and accessible via the submission site. Please log into your account, locate the manuscript record, and check for the action link "View Attachments". If this link does not appear, there are no attachment files.]

While revising your submission, please upload your figure files to the Preflight Analysis and Conversion Engine (PACE) digital diagnostic tool, https://pacev2.apexcovantage.com/. PACE helps ensure that figures meet PLOS requirements. To use PACE, you must first register as a user. Registration is free. Then, login and navigate to the UPLOAD tab, where you will find detailed instructions on how to use the tool. If you encounter any issues or have any questions when using PACE, please email PLOS at figures@plos.org. Please note that Supporting Information files do not need this step.

# Author comment: Author response to Decision Letter 0

**DOI:** 10.1371/journal.pone.0251889.r002

18 Mar 2021

Dear Editor, 

Dear reviewers,

We would like to thank you for the thoughtful and favorable reviews and the opportunity to revise our manuscript. We believe that our manuscript has benefitted considerably from the remarks and we hope that we addressed the mentioned issues to your satisfaction. Please also note that we made some minor changes in the text and minor changes in design, the latter to adapt to the journal style.

Below you can find our responses to the reviewer’s comments. Please also find attached our revised manuscript in two versions: with and without tracked changes. 

With our highest regards,

The authors

Comments to the Authors

REVIEWER 1: 

1. Abstract: nothing to add.

2. Introduction:

Specifying the difficulties of implementing clinical-radiologic rounds would be recommendable, especially considering these precise difficulties are the ones motivating the e-mail centric design of the study

Response: 

We thank the reviewer for this recommendation and modified the text accordingly:

However, clinical-radiologic rounds are often difficult to implement in the existing hospital structure, as rounds are often time-consuming and time schedules of staff can vary from day to day [6, 13].

3. Methods

Even if mentioned later in the section of Results, study population should be described in this section as well.

Response: 

The paragraphs about participants and patients are now found in the Methods section.

4. Results

It is stated that 39 of 49 total employees available participated in both rounds of the survey, however it is not stated if that 80% signify that there were 10 employees that dropped out between rounds of the survey, or if there were 10 employees who declined to take part of the study at all. If there was a 20% drop out between rounds, was there any registration for the reasons behind it?

Even though short-term feedback is defined from admission to discharge, no mention is made about of how long that time is, or which was the mean hospital stay of the patients included in the sample.

Response: 

The reviewer rightfully points out that it does not become clear, why and when the ten non-participants dropped out of the study. Initially all employees took part. Ten participants from the first round of the survey could no longer take part in the second round of the survey because they were no longer employed in the department or were on maternity leave. This is now specified in the Methods section:

“Initially, 49 of 49 employees participated in our survey. Ten of 49 (20%) participants dropped out because they were on maternity leave or were no longer employed in our department during the second survey round.“

The reviewer also refers to the missing information about the duration of hospital stay of patients. We added this information in the Methods section:

“The average length of stay in the hospital was 13.7±12.0 days.”

5. Discussion and Limitations

No mention is made from the possible selection bias from the voluntary participation in which the study design rests.

Response: 

The reviewer addresses the important issue of a selection bias. While we only took up the issue of a non-response bias in our limitations section, we now added the issue of a selection bias:

“The risk of a selection or a non-response bias is low, because all employees participated in our survey and the 10 non-participants during the second survey round were either on maternity leave or no longer employed in our department.”

REVIEWER 2:

1. The results are interesting, as it seems to make a difference between the training of physicians, radiographers and researchers, however, I would like to clarify who is part of each group, for example, in the group of physicians, are emergency physicians and neurologists included?, in the group of radiologists, are diagnostic radiologists and interventional neuroradiologists included?, etc.

Response: 

We thank the reviewer for pointing out this inaccuracy. All radiologists and radiographers in our department do diagnostic work and all but two senior radiologists and two senior radiographers also do interventional work. The latter two radiologists and two technicians have a long experience in neuro-interventions and stroke-therapy. Workstations alternate regularly, often on a daily basis. Emergency physicians, neurologists, and anaesthesiologists are not employed by our department and were therefore not part of this survey.

“All radiologists and radiographers in our department do diagnostic work (mainly computed tomography, magnet resonance tomography, and angiography) and all but two senior radiologists and two senior radiographers also do interventional work. The latter two radiologists and two technicians have a long experience in neuro-interventions and stroke-therapy. Workstations alternate regularly, often on a daily basis. Emergency physicians, neurologists, and anaesthesiologists have important functions during interventional stroke therapy, but are not employed by our department of Neuroradiology and are consequently not part of this survey.”

2. Could you say if scientists and researchers mean the same thing in your writing?

Response: 

Using “scientists” and “researchers” was an unnecessarily awkward inacurracy. We now consistently refer to “researchers”.

3. Could you define the meaning of PACS? This way anyone who reads your article will know what you mean.

Response: 

We thank the reviewer for pointing out this inaccuracy. We now spelled out this abbreviation in the text: Picture Archiving and Communication System (PACS).

Attachment Submitted filename: Response to Reviewers.docx Click here for additional data file.

# Peer review: Decision Letter 1

**DOI:** 10.1371/journal.pone.0251889.r003

**Authors:** Miguel A. Barboza

5 May 2021

Work satisfaction among neuroradiology staff after receiving follow up reports of thrombectomy stroke patients

PONE-D-21-03388R1

Dear Dr. Nikoubashman,

We’re pleased to inform you that your manuscript has been judged scientifically suitable for publication and will be formally accepted for publication once it meets all outstanding technical requirements.

Within one week, you’ll receive an e-mail detailing the required amendments. When these have been addressed, you’ll receive a formal acceptance letter and your manuscript will be scheduled for publication.

An invoice for payment will follow shortly after the formal acceptance. To ensure an efficient process, please log into Editorial Manager at http://www.editorialmanager.com/pone/, click the 'Update My Information' link at the top of the page, and double check that your user information is up-to-date. If you have any billing related questions, please contact our Author Billing department directly at authorbilling@plos.org.

If your institution or institutions have a press office, please notify them about your upcoming paper to help maximize its impact. If they’ll be preparing press materials, please inform our press team as soon as possible -- no later than 48 hours after receiving the formal acceptance. Your manuscript will remain under strict press embargo until 2 pm Eastern Time on the date of publication. For more information, please contact onepress@plos.org.

Kind regards,

Miguel A. Barboza, MD, MSc

Academic Editor

PLOS ONE

Additional Editor Comments (optional):

Reviewers' comments:

Reviewer's Responses to Questions

**Comments to the Author**

1. If the authors have adequately addressed your comments raised in a previous round of review and you feel that this manuscript is now acceptable for publication, you may indicate that here to bypass the “Comments to the Author” section, enter your conflict of interest statement in the “Confidential to Editor” section, and submit your "Accept" recommendation.

Reviewer #2: All comments have been addressed

**********

2. Is the manuscript technically sound, and do the data support the conclusions?

The manuscript must describe a technically sound piece of scientific research with data that supports the conclusions. Experiments must have been conducted rigorously, with appropriate controls, replication, and sample sizes. The conclusions must be drawn appropriately based on the data presented. 

Reviewer #2: Yes

**********

3. Has the statistical analysis been performed appropriately and rigorously? 

Reviewer #2: Yes

**********

4. Have the authors made all data underlying the findings in their manuscript fully available?

The PLOS Data policy requires authors to make all data underlying the findings described in their manuscript fully available without restriction, with rare exception (please refer to the Data Availability Statement in the manuscript PDF file). The data should be provided as part of the manuscript or its supporting information, or deposited to a public repository. For example, in addition to summary statistics, the data points behind means, medians and variance measures should be available. If there are restrictions on publicly sharing data—e.g. participant privacy or use of data from a third party—those must be specified.

Reviewer #2: Yes

**********

5. Is the manuscript presented in an intelligible fashion and written in standard English?

PLOS ONE does not copyedit accepted manuscripts, so the language in submitted articles must be clear, correct, and unambiguous. Any typographical or grammatical errors should be corrected at revision, so please note any specific errors here.

Reviewer #2: Yes

**********

6. Review Comments to the Author

Please use the space provided to explain your answers to the questions above. You may also include additional comments for the author, including concerns about dual publication, research ethics, or publication ethics. (Please upload your review as an attachment if it exceeds 20,000 characters)

Reviewer #2: The authors have responded to all comments made in the first round, this article is now acceptable for publication.

**********

7. PLOS authors have the option to publish the peer review history of their article (what does this mean?). If published, this will include your full peer review and any attached files.

If you choose “no”, your identity will remain anonymous but your review may still be made public.

**Do you want your identity to be public for this peer review?** For information about this choice, including consent withdrawal, please see our Privacy Policy.

Reviewer #2: No

# Editor report: Acceptance letter

**DOI:** 10.1371/journal.pone.0251889.r004

**Authors:** Miguel A. Barboza

11 May 2021

PONE-D-21-03388R1 

Work satisfaction among neuroradiology staff after receiving follow up reports of thrombectomy stroke patients 

Dear Dr. Nikoubashman:

I'm pleased to inform you that your manuscript has been deemed suitable for publication in PLOS ONE. Congratulations! Your manuscript is now with our production department. 

If your institution or institutions have a press office, please let them know about your upcoming paper now to help maximize its impact. If they'll be preparing press materials, please inform our press team within the next 48 hours. Your manuscript will remain under strict press embargo until 2 pm Eastern Time on the date of publication. For more information please contact onepress@plos.org.

If we can help with anything else, please email us at plosone@plos.org. 

Thank you for submitting your work to PLOS ONE and supporting open access. 

Kind regards, 

PLOS ONE Editorial Office Staff

on behalf of

Dr. Miguel A. Barboza 

Academic Editor

PLOS ONE
